# Supplementary figures and images for: An objective criterion to evaluate sequence-similarity networks helps in dividing the protein family sequence space
Source: PLoS Comput Biol. 2023 Aug 16;19(8):e1010881. doi: 10.1371/journal.pcbi.1010881 (PMC10461819; doi:10.1371/journal.pcbi.1010881)

A

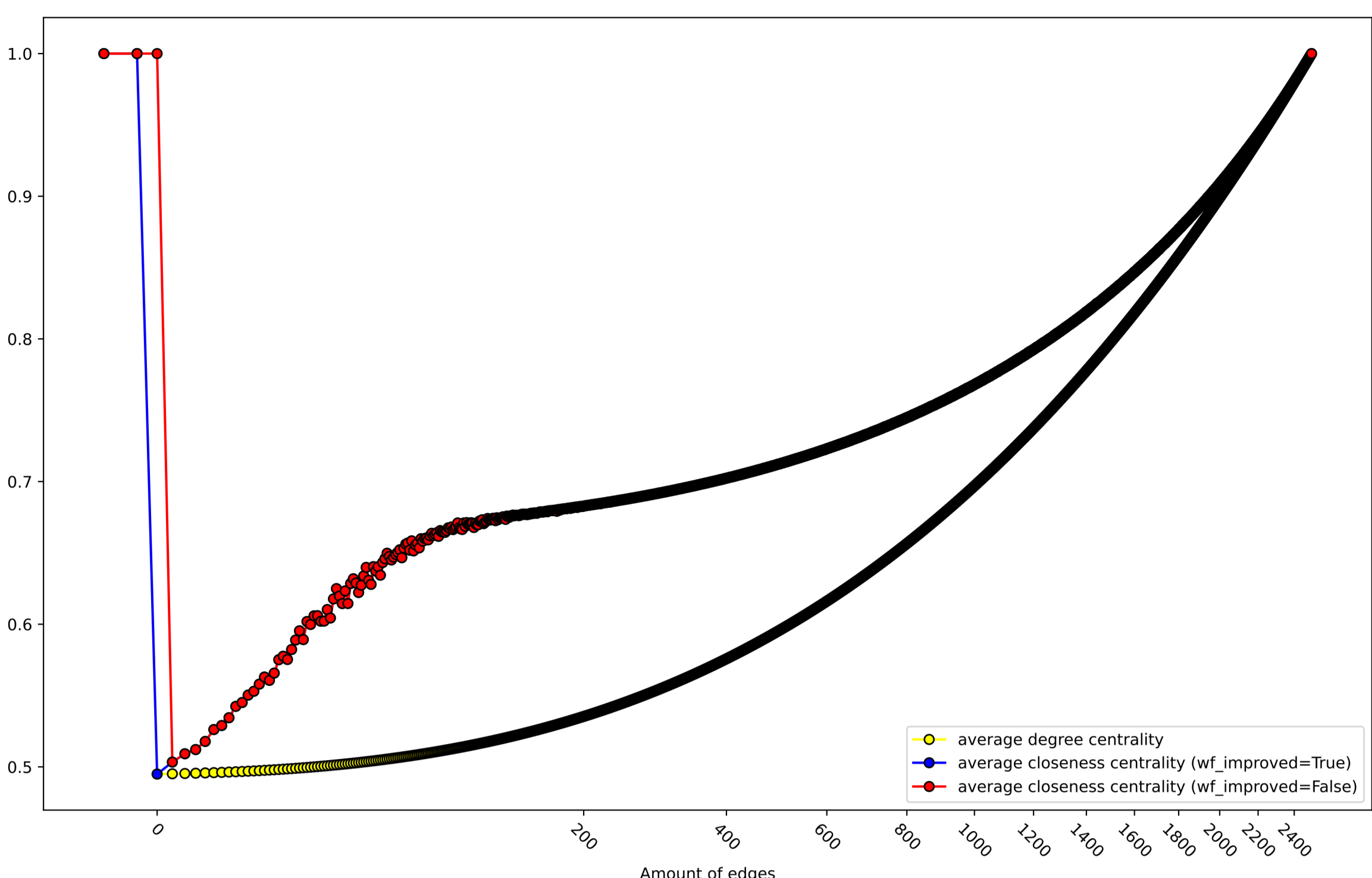

B

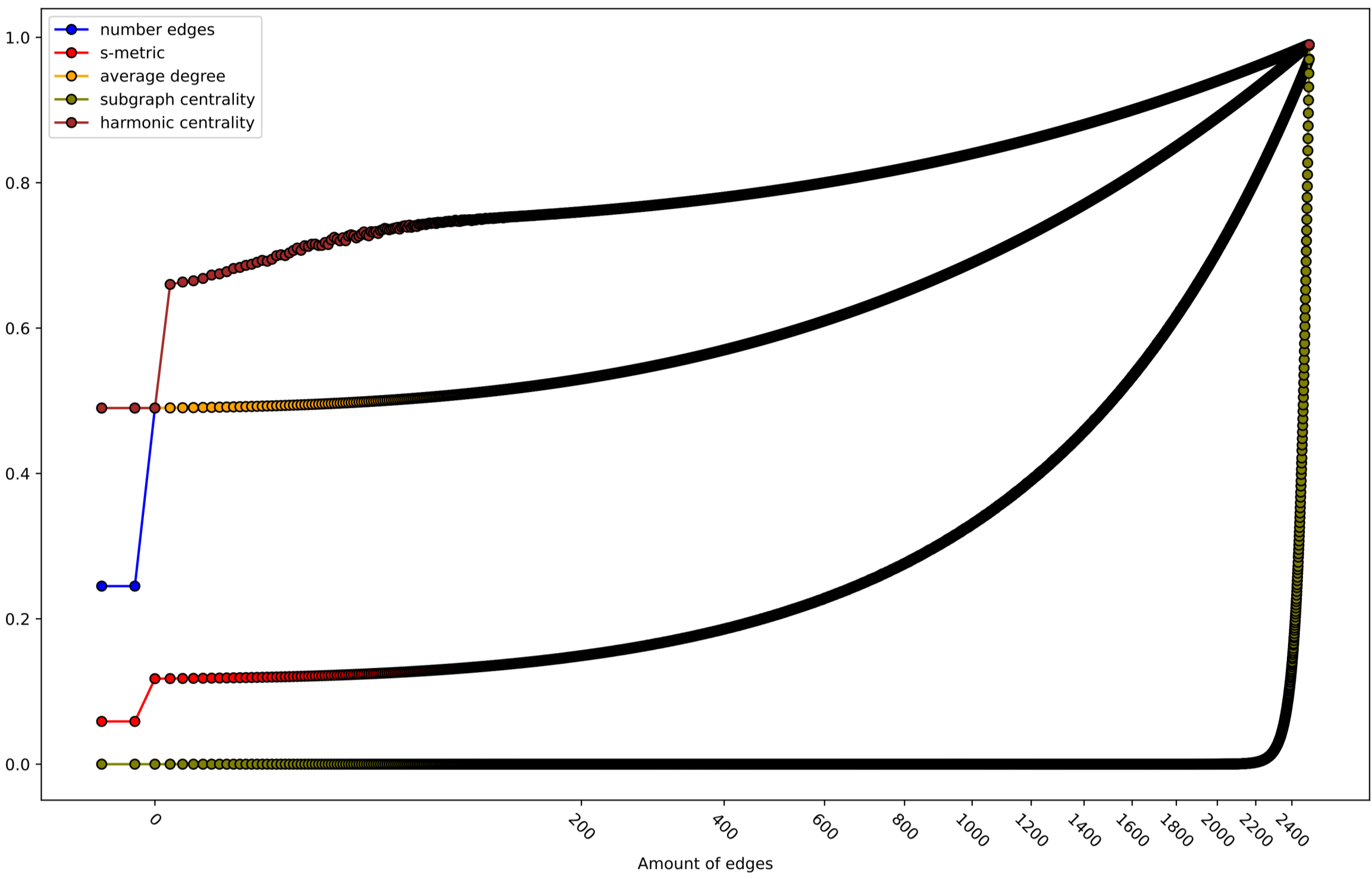

C

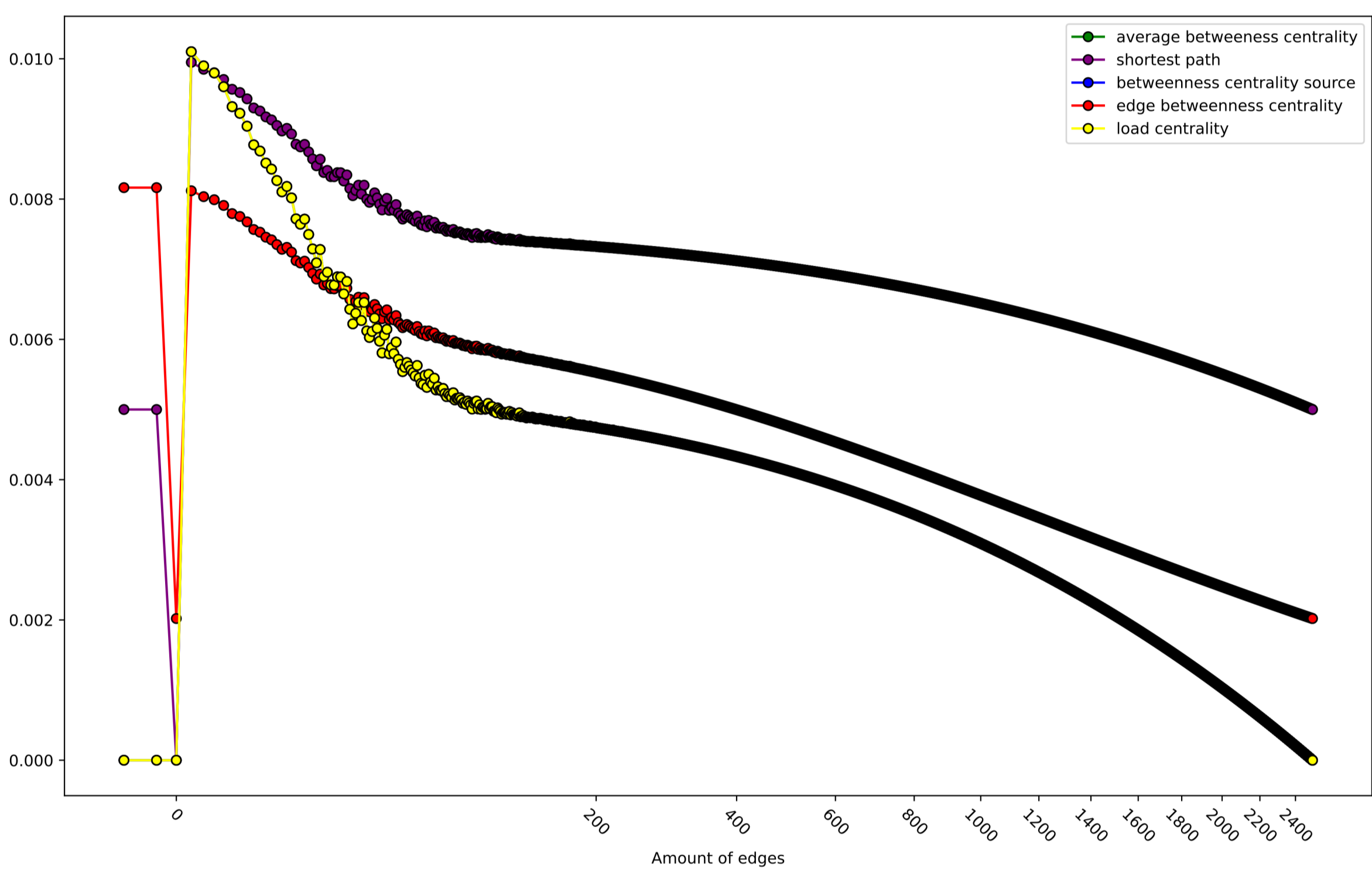

D

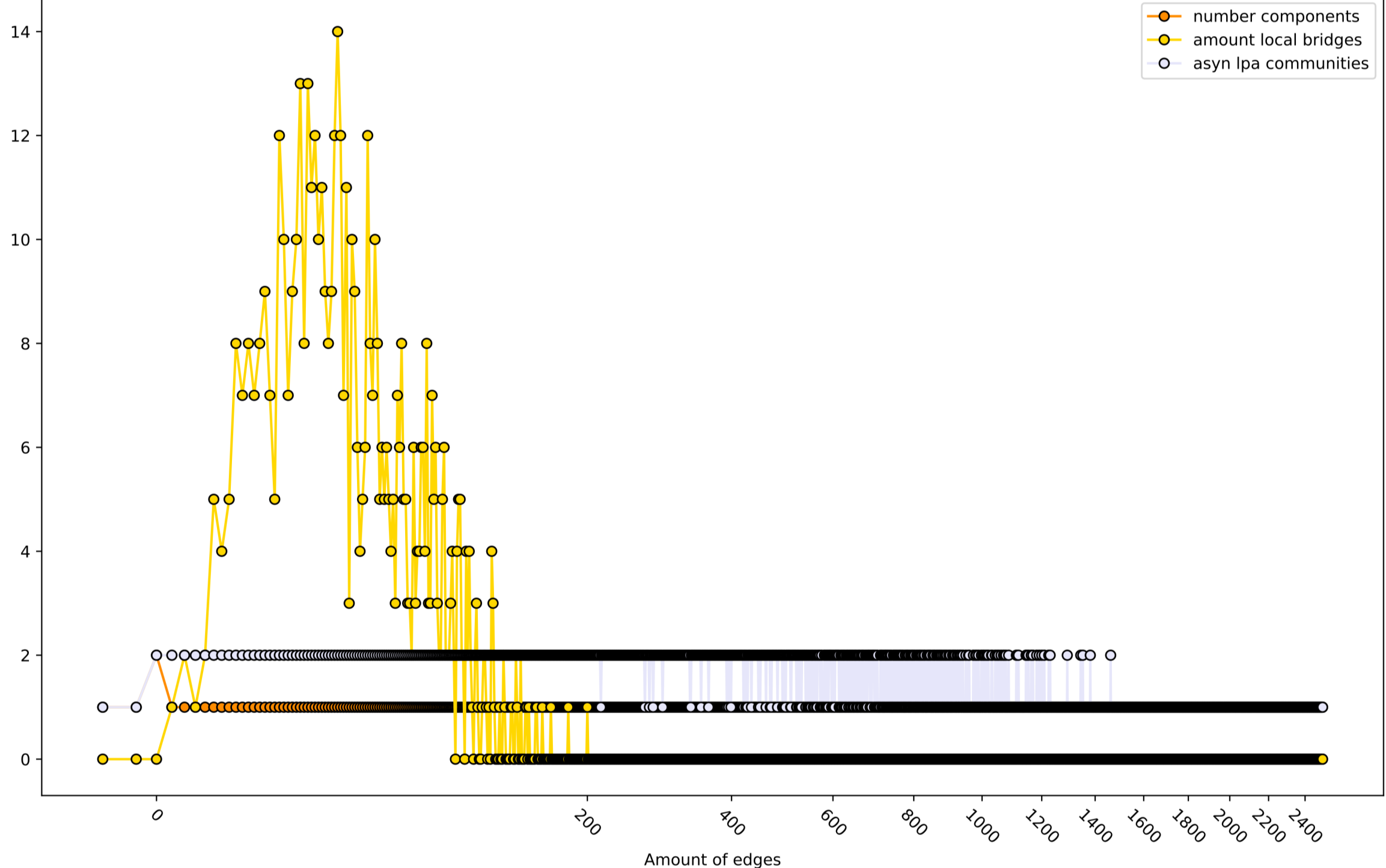

E

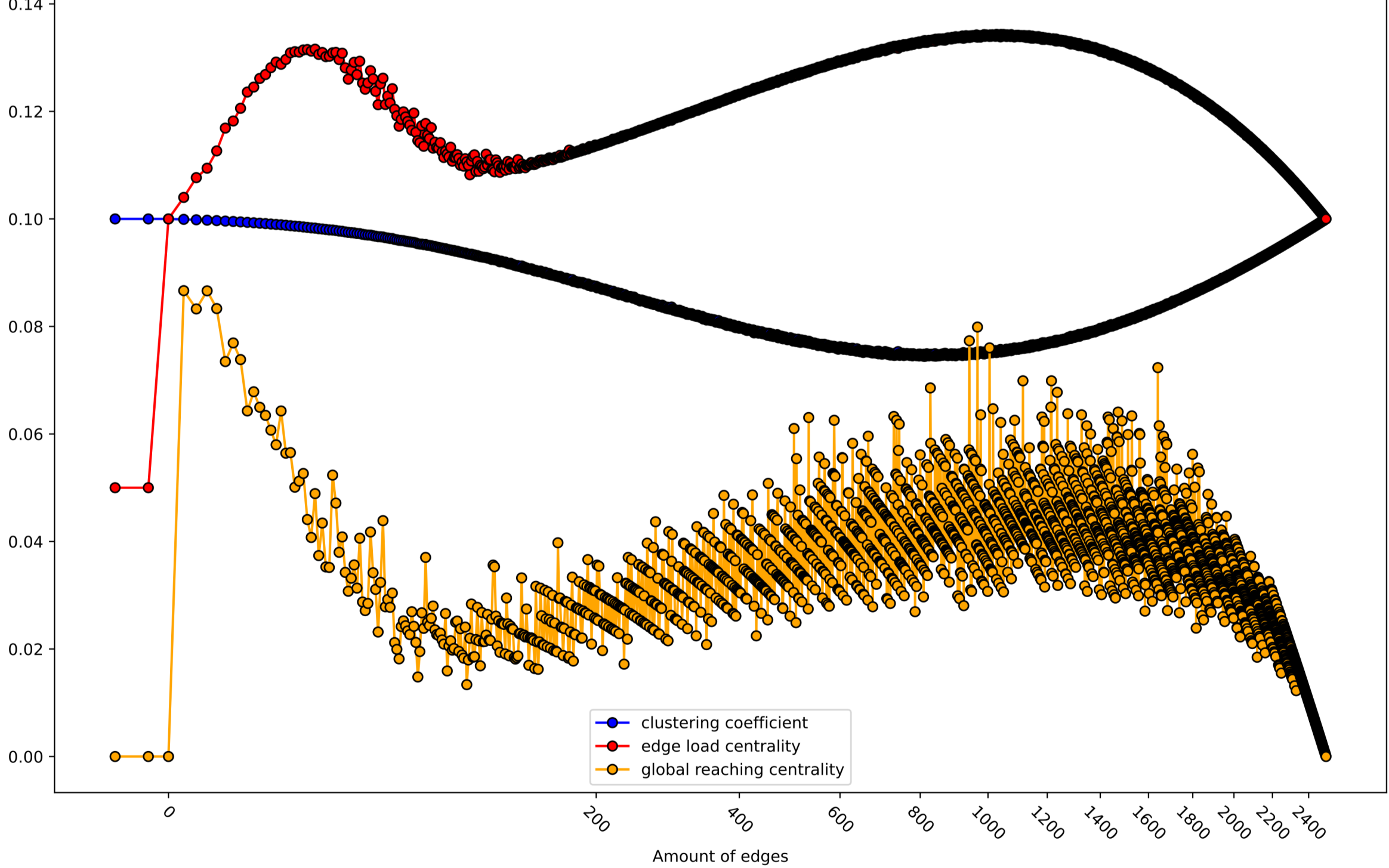

F

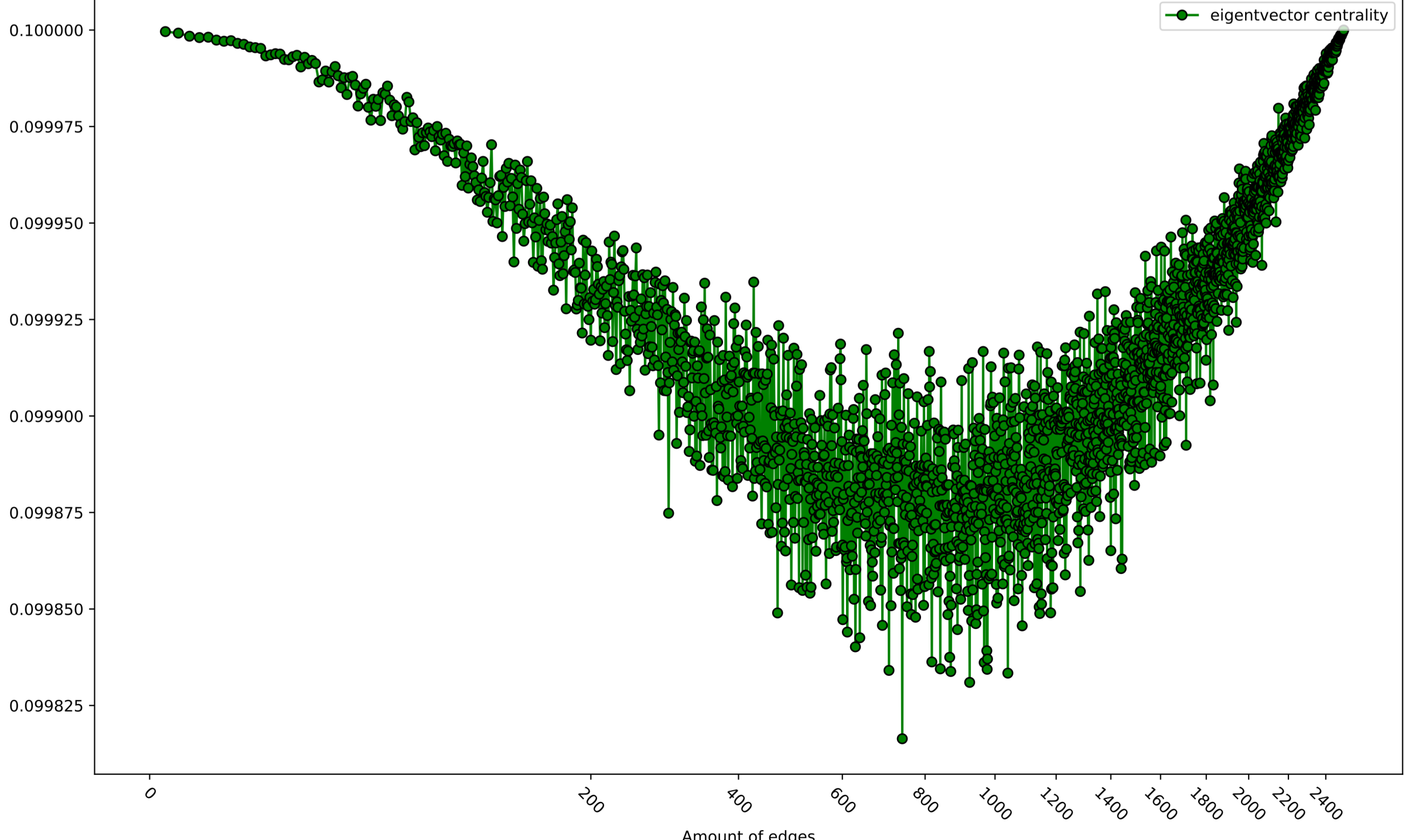

Supplement: S1 Fig — A) Closeness and degree centrality both favour more edges over less, and both networks combined with zero connections score worse than a network with one connection. The adjustment to score these networks better has been performed in this work. B) The number of edges, the s-metric, the average degree, subgraph centrality and harmonic centrality show a monotonous behaviour. The more edges a component possesses, the better the score. This means that one perfect network scores worse than two perfect networks with one connection. C) Betweenness and similar metrics score in an inverted way to closeness in A). Based on this behaviour it cannot be decided which metric is more suitable. A decision had to be made based on the performance with single nodes, as discussed in the manuscript. D) The amount of bridges, components and communities does not show a quantitative enough behaviour. Many instances are scored with a value of one, making it not possible to discriminate between better or worse networks. E) and F) show the behaviour of the remaining four criteria. The eigenvector centrality had to be plotted separately, without the first three data points, due to differences in scale between these data points. All four metrics show a behaviour which might favour less edges over more edges at some point in the data set, making their behaviour not desirable. Parameters may have been scaled for display purposes. (PDF) [file pcbi.1010881.s005.pdf]

Evalue distribution in relation to identity percentage for PL26

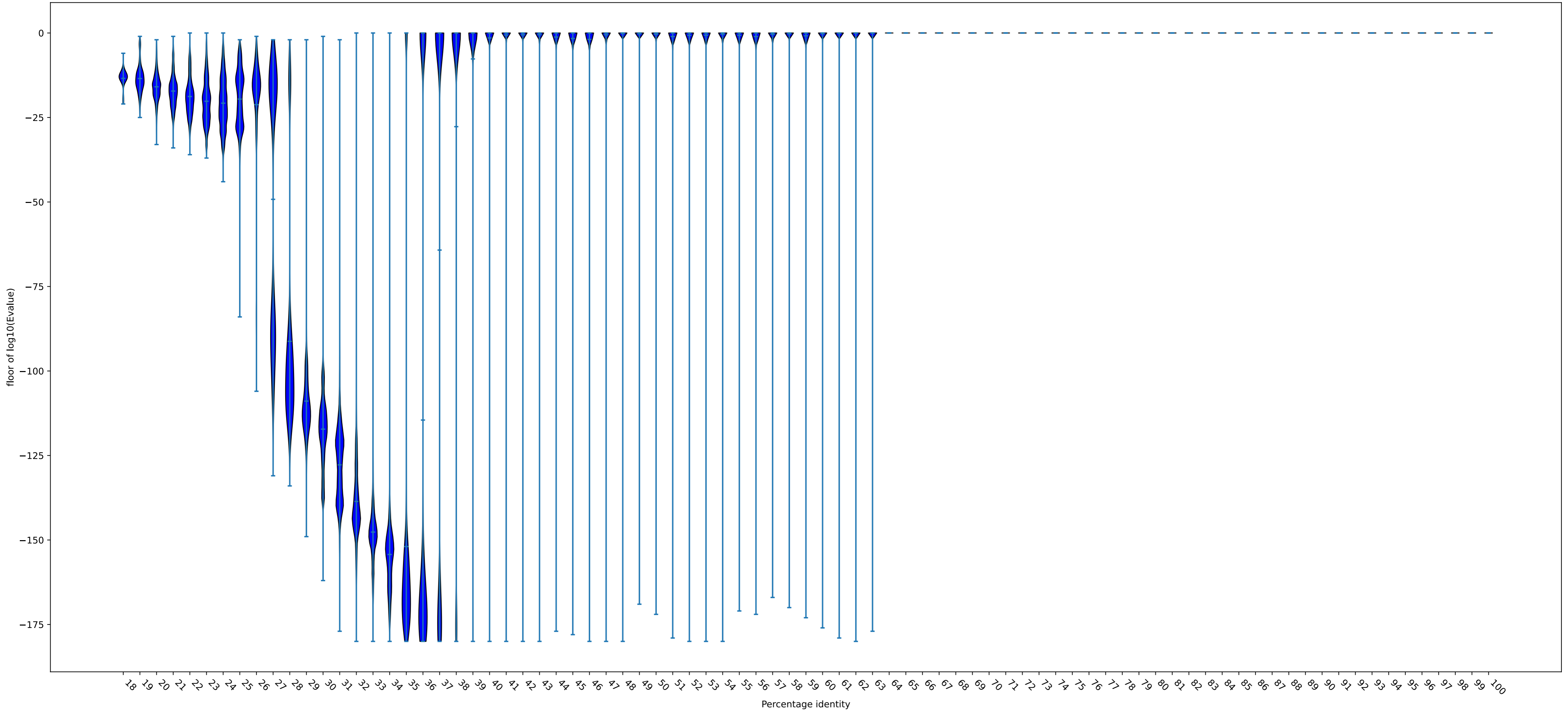

Supplement: S5 Fig — With 32% identity between two sequences the first evalue of zero was obtained. With 64% identity between two sequences all evalues correspond to zero. (PDF) [file pcbi.1010881.s009.pdf]

A

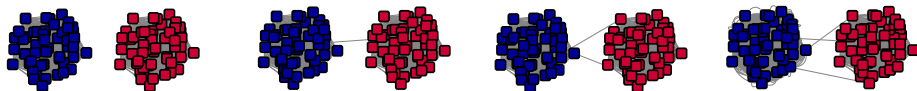

B

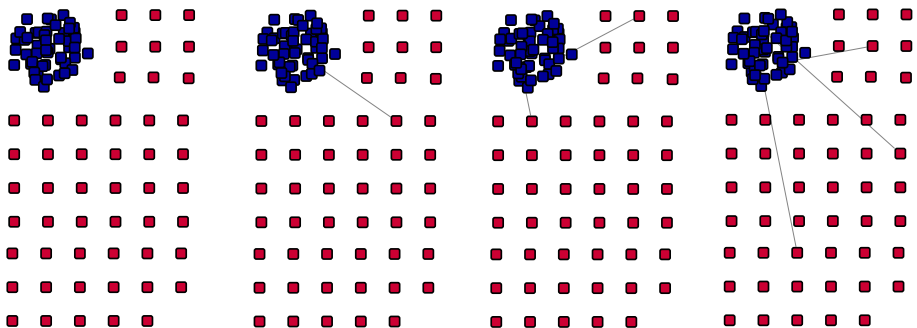

C

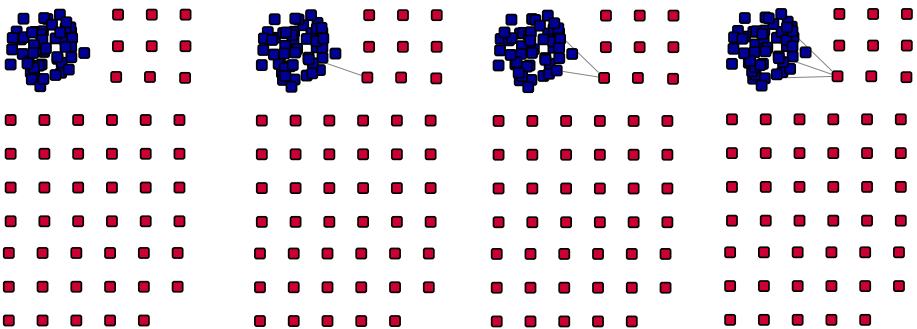

Supplement: S6 Fig — A) One network set, consisting out of two networks, which are connected with increasing amounts of edges. B) Special case #1: one network is connected to a network of singles. Each single node is connected first by only one connection, and not random, preventing a second connection before all nodes are connected. C) Special case #2: one network is connected to a network of singles. Each single node is connected first to all nodes in the main network. (PDF) [file pcbi.1010881.s010.pdf]

A

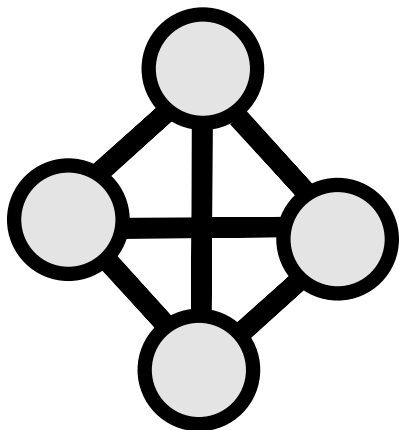

B

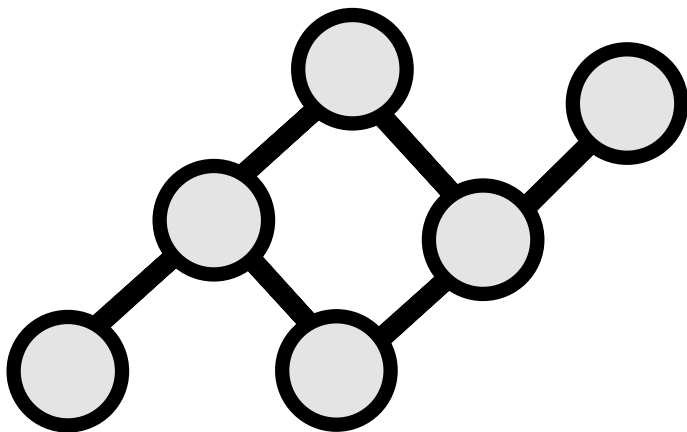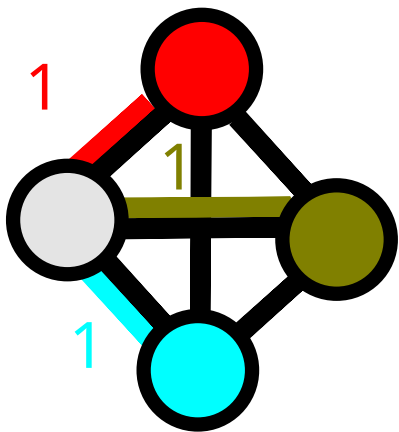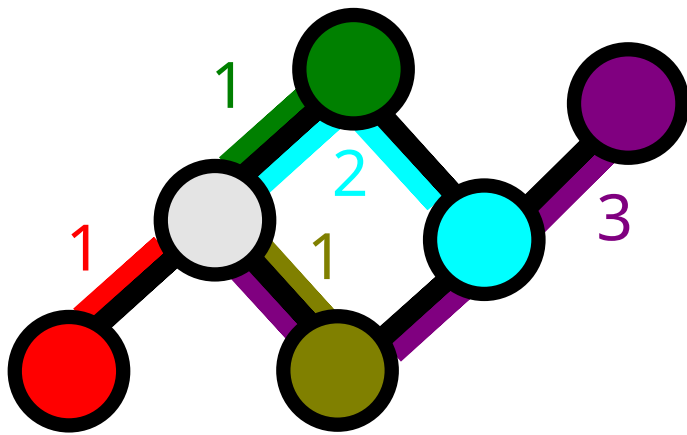

Supplement: S7 Fig — In A) a perfect network is displayed. From the example of the grey node, all distances to other nodes (red, cyan, beige) is one. B) an imperfect network is displayed. From the grey node three distances are one (red, green, beige), one distance is two (cyan), and one three (purple). The average of these paths is used as the closeness of the whole network. (PDF) [file pcbi.1010881.s011.pdf]

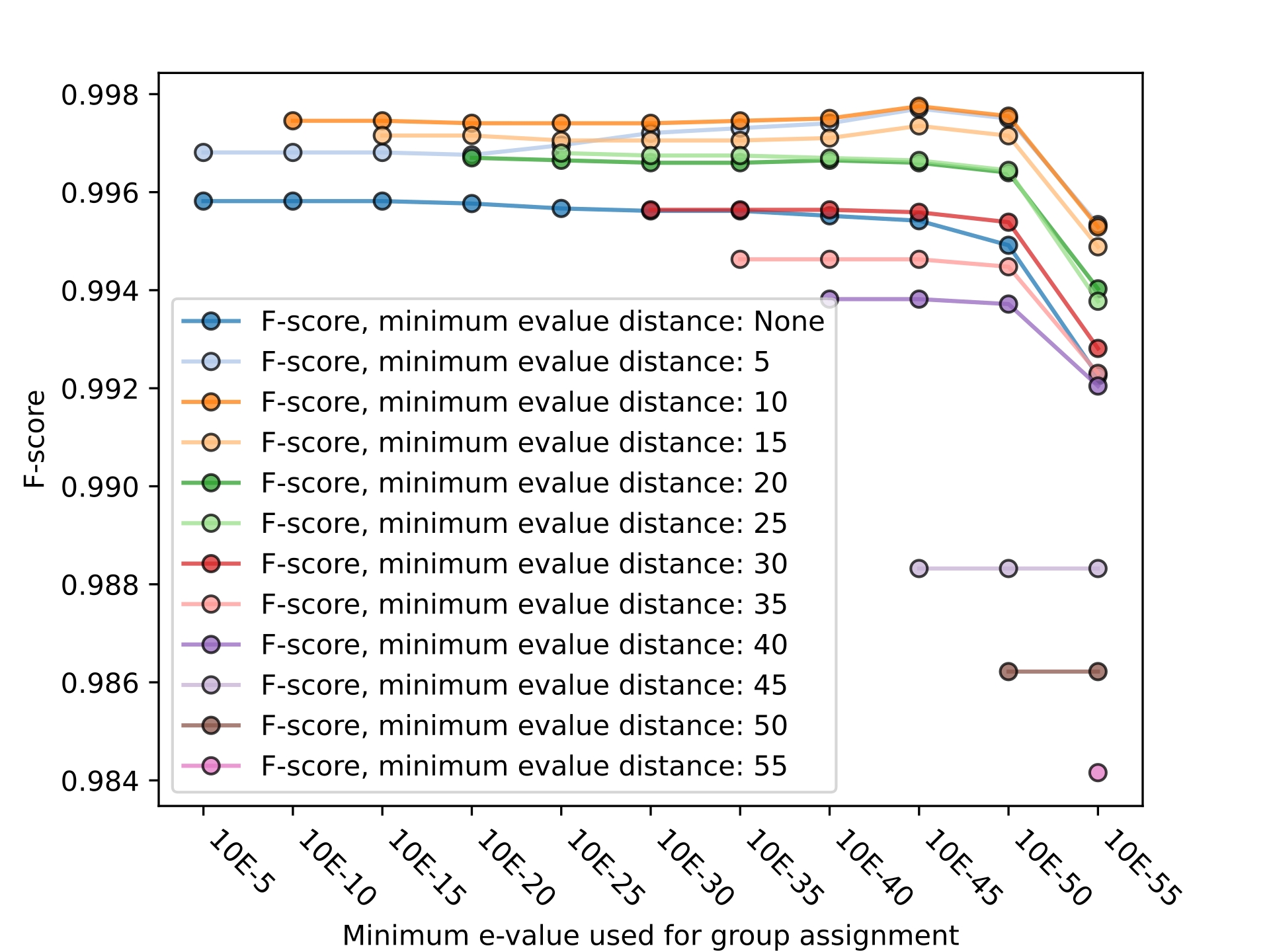

Supplement: S8 Fig — The distance to the next best HMM hit was also considered, but choosing no difference (first line in legend) or choosing a minimum of 10−5 e-value difference to the second best hit (second line in legend) did not make any difference in this case (graphs overlap). (JPG) [file pcbi.1010881.s012.jpg]

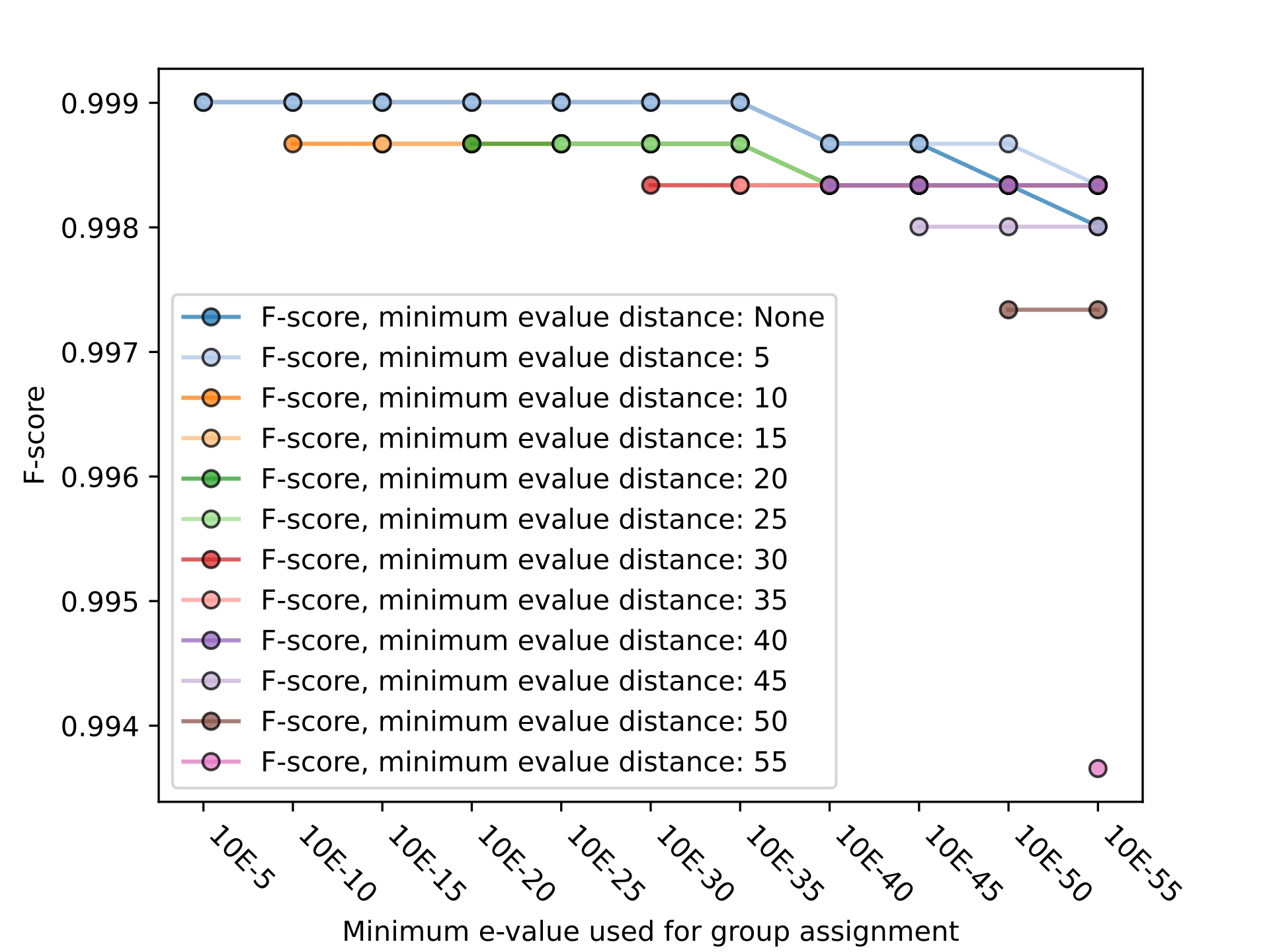

Supplement: S9 Fig — In addition, the distance to the next best HMM hit has to be considered, since requiring a minimum of 10E-10 between the best HMM hit and the second best HMM hit gave the best result. (JPG) [file pcbi.1010881.s013.jpg]
