# Supplementary material for: An objective criterion to evaluate sequence-similarity networks helps in dividing the protein family sequence space
Source: PLoS Comput Biol. 2023 Aug 16;19(8):e1010881. doi: 10.1371/journal.pcbi.1010881 (PMC10461819; doi:10.1371/journal.pcbi.1010881)
Supplement: S2 Fig — At 10−29 the first network of 699 nodes contains 32 experimentally validated enzymes, the second network of 248 contains 18, and the last network does not contain any characterized enzymes. The bottom row summarizes the amount of protein sequences not in a subfamily at the respective evalue. (PDF) [file pcbi.1010881.s006.pdf]

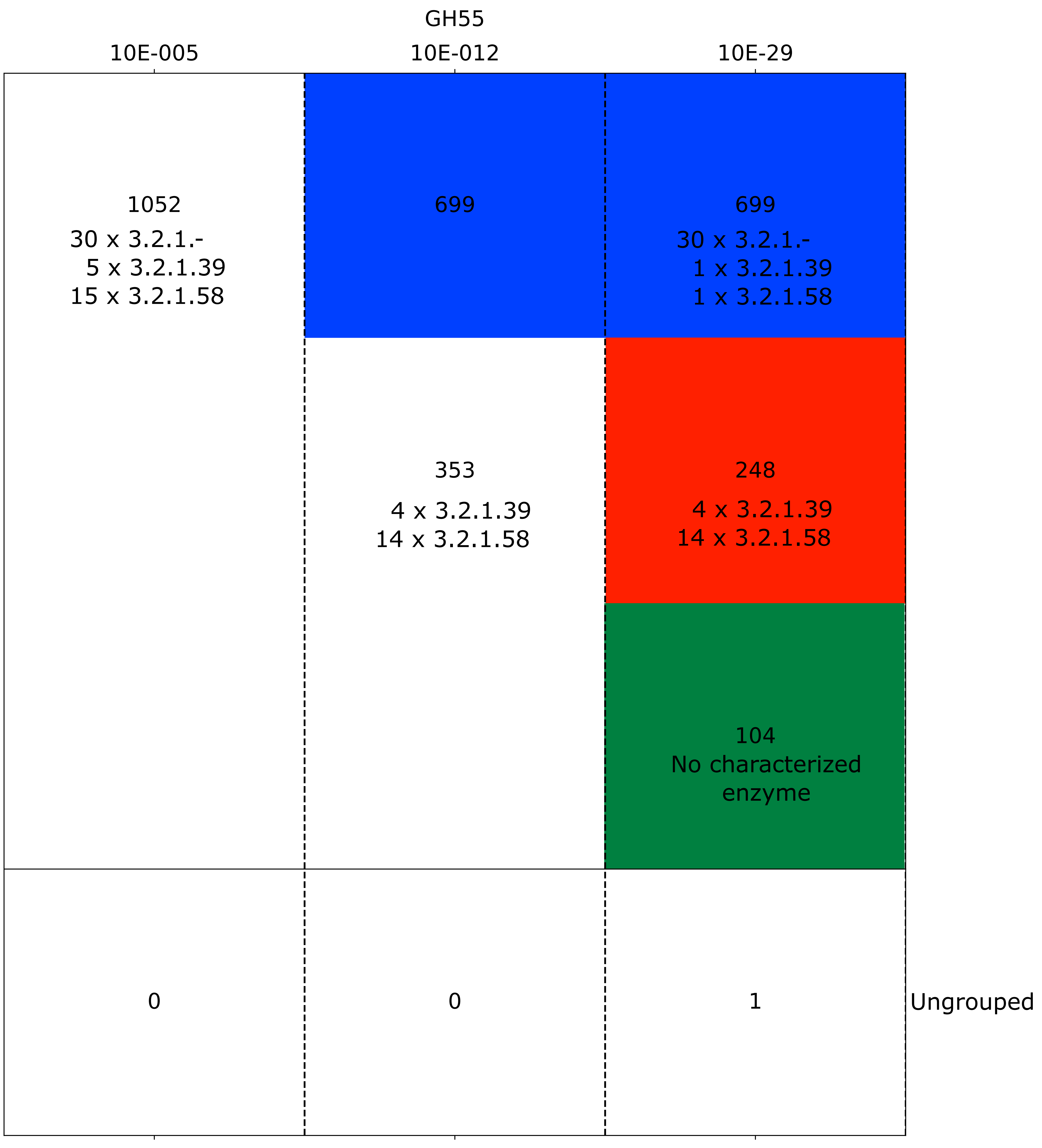
GH55
10E-005
10E-012
10E-29
1052
30 x 3.2.1.-
5 x 3.2.1.39
15 x 3.2.1.58
699
699
30 x 3.2.1.-
1 x 3.2.1.39
1 x 3.2.1.58
353
4 x 3.2.1.39
14 x 3.2.1.58
248
4 x 3.2.1.39
14 x 3.2.1.58
104
No characterized enzyme
0
0
1
Ungrouped
