# Supplementary material for: An objective criterion to evaluate sequence-similarity networks helps in dividing the protein family sequence space
Source: PLoS Comput Biol. 2023 Aug 16;19(8):e1010881. doi: 10.1371/journal.pcbi.1010881 (PMC10461819; doi:10.1371/journal.pcbi.1010881)
Supplement: S3 Fig — (PDF) [file pcbi.1010881.s007.pdf]

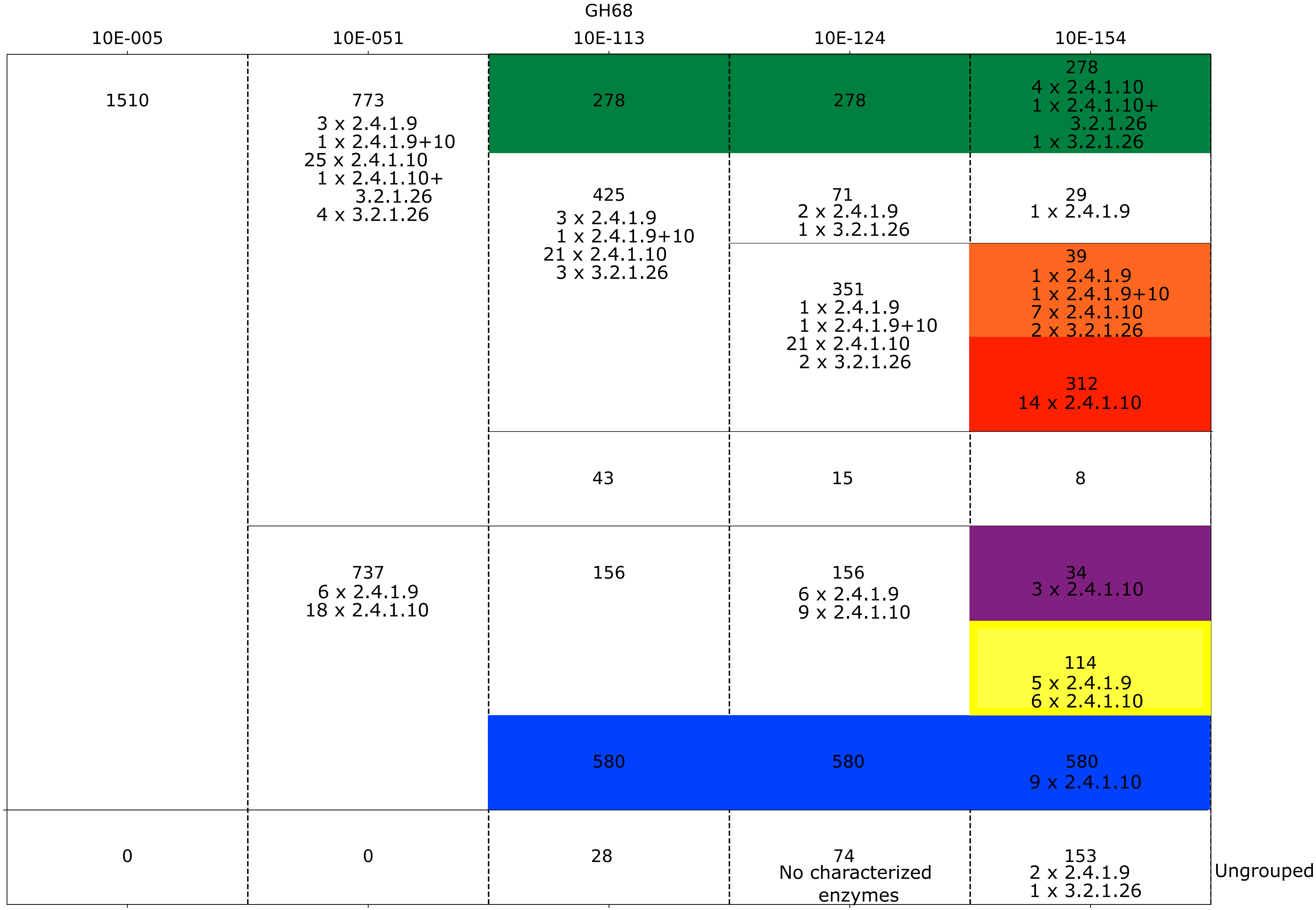
GH68
10E-005
10E-051
10E-113
10E-124
10E-154
1510
773
3 x 2.4.1.9
1 x 2.4.1.9+10
25 x 2.4.1.10
1 x 2.4.1.10+ 3.2.1.26
4 x 3.2.1.26
278
278
278
4 x 2.4.1.10
1 x 2.4.1.10+ 3.2.1.26
1 x 3.2.1.26
425
3 x 2.4.1.9
1 x 2.4.1.9+10
21 x 2.4.1.10
3 x 3.2.1.26
71
2 x 2.4.1.9
1 x 3.2.1.26
29
1 x 2.4.1.9
39
1 x 2.4.1.9
1 x 2.4.1.9+10
7 x 2.4.1.10
2 x 3.2.1.26
351
1 x 2.4.1.9
1 x 2.4.1.9+10
21 x 2.4.1.10
2 x 3.2.1.26
312
14 x 2.4.1.10
43
15
8
737
6 x 2.4.1.9
18 x 2.4.1.10
156
156
6 x 2.4.1.9
9 x 2.4.1.10
34
3 x 2.4.1.10
114
5 x 2.4.1.9
6 x 2.4.1.10
580
580
580
9 x 2.4.1.10
0
0
28
74
No characterized enzymes
153
2 x 2.4.1.9
1 x 3.2.1.26
Ungrouped
