# Supplementary material for: An objective criterion to evaluate sequence-similarity networks helps in dividing the protein family sequence space
Source: PLoS Comput Biol. 2023 Aug 16;19(8):e1010881. doi: 10.1371/journal.pcbi.1010881 (PMC10461819; doi:10.1371/journal.pcbi.1010881)
Supplement: S4 Fig — (PDF) [file pcbi.1010881.s008.pdf]

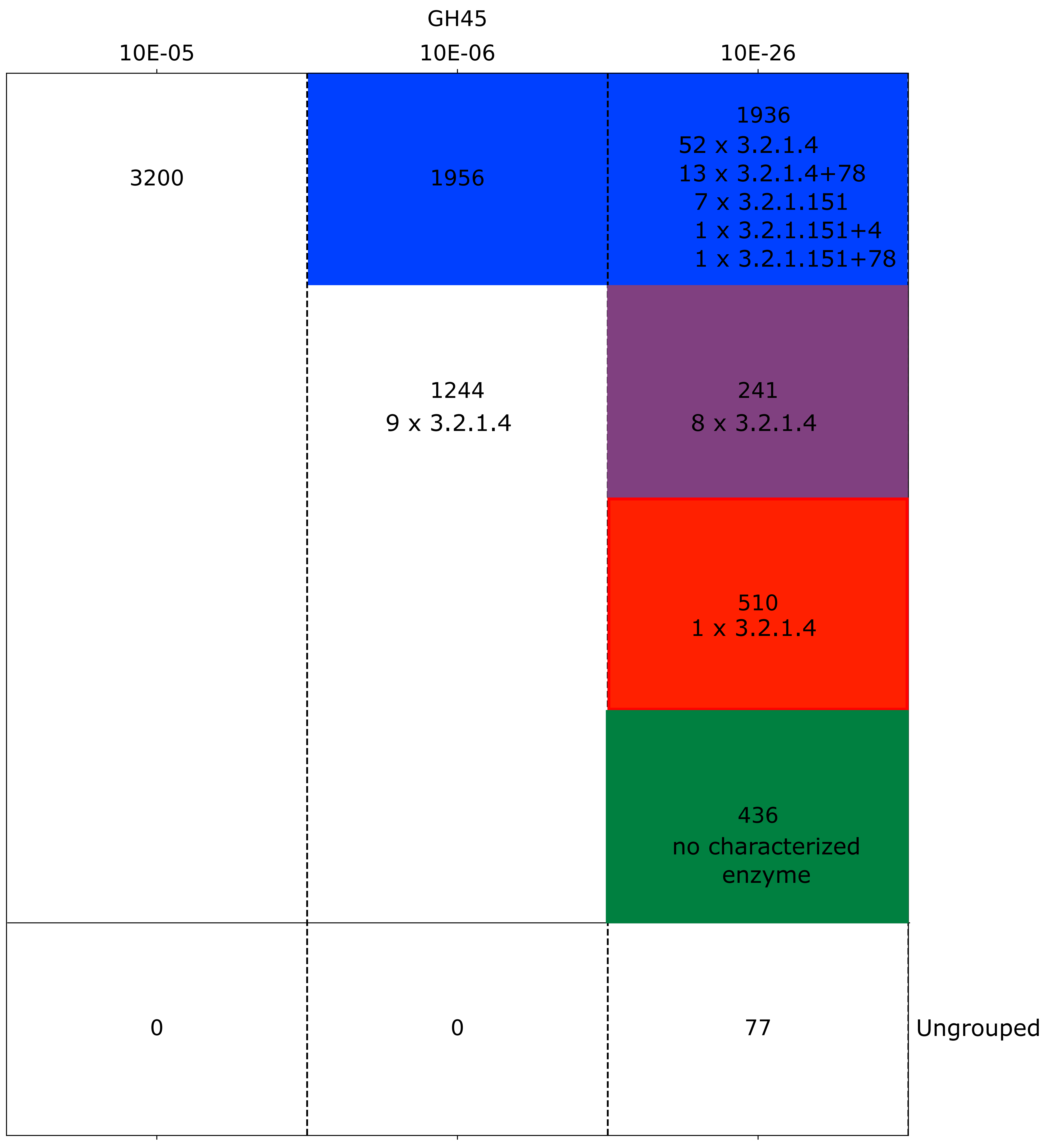
GH45
10E-05
10E-06
10E-26
3200
1956
1936
52 x 3.2.1.4
13 x 3.2.1.4+78
7 x 3.2.1.151
1 x 3.2.1.151+4
1 x 3.2.1.151+78
1244
9 x 3.2.1.4
241
8 x 3.2.1.4
510
1 x 3.2.1.4
436
no characterized enzyme
0
0
77
Ungrouped
