# Supplementary material for: An objective criterion to evaluate sequence-similarity networks helps in dividing the protein family sequence space
Source: PLoS Comput Biol. 2023 Aug 16;19(8):e1010881. doi: 10.1371/journal.pcbi.1010881 (PMC10461819; doi:10.1371/journal.pcbi.1010881)
Supplement: S10 Fig — For GH68, both groupings at 10E-51 and 10E-154 are displayed. For the SFLD families, pre-computed groups were used. While in the PCA plot for GH55 the three subfamilies are visible, this gets ambigious in the MDS plot or for GH68, and indistinguishable for the SFLD families. (PDF) [file pcbi.1010881.s014.pdf]

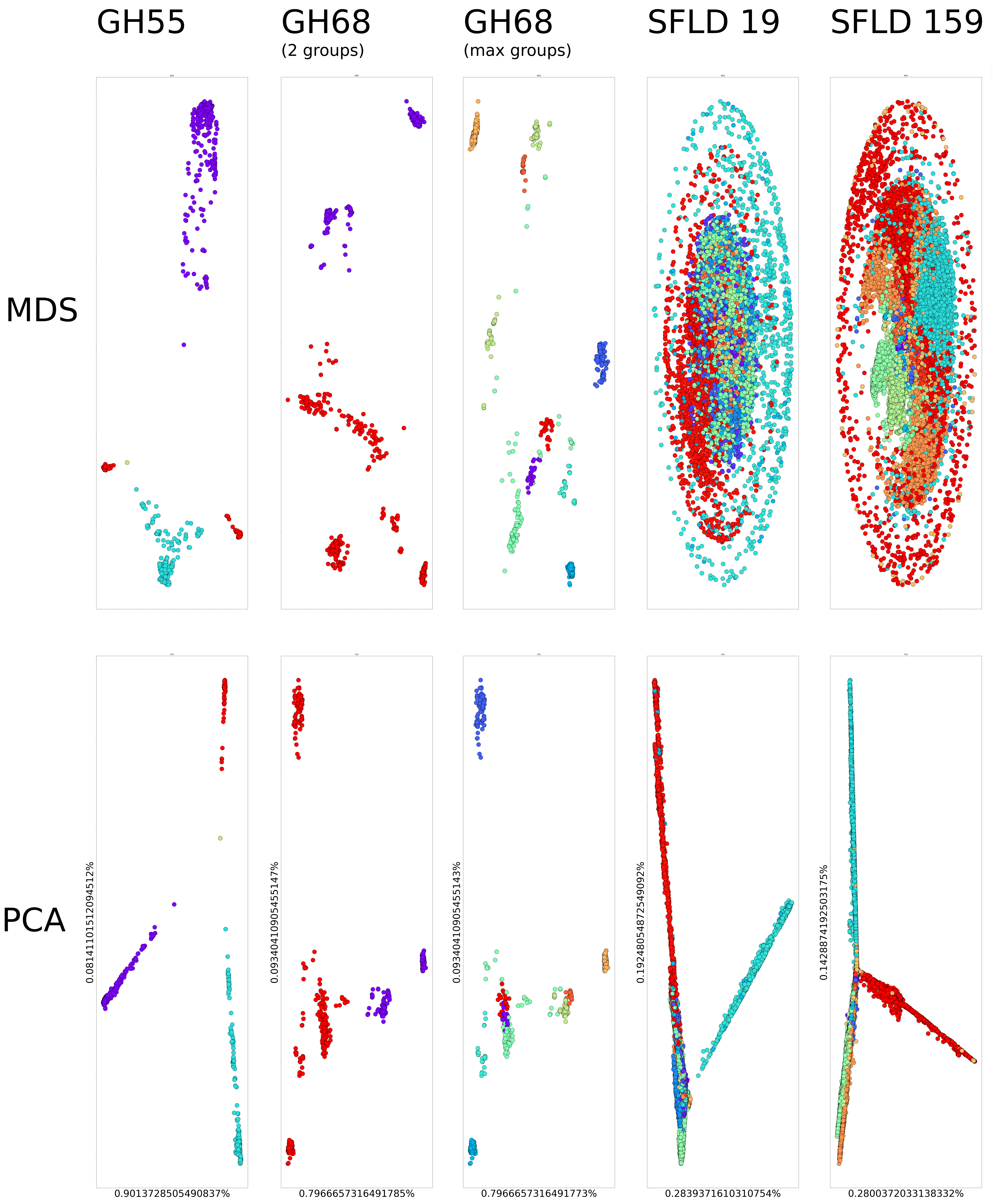
GH55
GH68
(2 groups)
GH68
(max groups)
SFLD 19
SFLD 159
MDS
PCA
0.08141101512094512%
0.9013728505490837%
0.09340410905455147%
0.7966657316491785%
0.09340410905455143%
0.7966657316491773%
0.19248054872549092%
0.2839371610310754%
0.1428874192503175%
0.2800372033138332%
