# Supplementary material for: An objective criterion to evaluate sequence-similarity networks helps in dividing the protein family sequence space
Source: PLoS Comput Biol. 2023 Aug 16;19(8):e1010881. doi: 10.1371/journal.pcbi.1010881 (PMC10461819; doi:10.1371/journal.pcbi.1010881)
Supplement: Attachment — Submitted filename: Reviewer comments for PCOMPBIOL.docx [file pcbi.1010881.s015.docx]

Reviewer comments for PCOMPBIOL-D-22-00646

“An objective criterion to evaluate sequence-similarity networks helps in dividing the protein family sequence space”

Protein functional annotation is still dependent on expert curation for the most accurate delineation. The attached manuscript describes a novel use of closenesss centrality, a well-known measure in network analysis, to analyze sequence similarity networks (SSNs). The authors rather exhaustively test this criterion on artificial data, known protein families with existing network analyses, and novel families that have not yet been analyzed by SSN. Despite lacking the more rigorous evolutionary foundation of phylogenetic analysis, the authors make a strong case that SSNs are a more performant method for accounting for the deluge of sequencing data. This work is novel, has a strong premise, and is an innovation combination of well-established databases and graph network analyses. Nonetheless, there are a few points that should be addressed in order for this work to be published. I have organized them into major and minor points below. I recommend that this work be published after minor revision.

**Major Points**

1) A major strength of SSNs is their relatively perfomance with respect to phylogenetic analyses and other types of dimension reduction. Similarly, a stated value of the closeness centrality criterion is that it is faster than manually testing e-value cutoffs. I would like to see more explicit benchmarking data for the artificial data, known families, and novel families. If run times are neglible a statement in the methods to that effect would be appropriate. A table, either in the supplementary material or main text elaborating on this performance and how it varies with respect to family size would also work.
2) The sizes and cutoffs of the various families being considered are difficult to follow in the text. It would provide clarity to make a summary table that elaborated on the families considered, their accessions in CAZY (where appropriate), the cutoffs determined by closeness centrality, and any cutoffs determined previousy in other works.
3) The current figures are not publication ready. Generally, even at full size, it is difficult to resolve edge nodes, axis labels are illegible. Insofar as a principal point of the manuscript is the relative ease of SSNs and their visualization, the complexity of these figures, both individually and collectively, works against this point. I would recommend a) making a schematic representation of the artificial data results and either making it comparative with the “real” data, then having a more legible version of the closeness vs. cutoff graph. I am also not certain the full set of these figures is necessary in the main text, some could be moved to supplementary.
4) I would like some more elaboration (perhaps in the form of a figure) of an example where the closeness criterion is separating into functional vs. structural groups

**Minor Points**

1) There are numerous grammatical errors. A close reading for line edits is necessary. I have provided some corrections below but the list is not exhaustive.

P2,L50:	Determining => Determine

P2,L52:	“Cazbohydrate”

P2,L54:	“And guide” => “And to guide”

P2,L99:	“build hypothesis” => “build hypotheses” or “build a hypothesis”

P6,L146:”consisted in network collections” => “consisted of network collections”

P6,L155: Not clear what i) ii) iii) iv) are indicating in this paragraph

P11,L334: “Such situation might” => “Such a situation might”

P12,L351: “group has not been seen” => “group had not been seen”
