# Supplementary material for: An objective criterion to evaluate sequence-similarity networks helps in dividing the protein family sequence space
Source: PLoS Comput Biol. 2023 Aug 16;19(8):e1010881. doi: 10.1371/journal.pcbi.1010881 (PMC10461819; doi:10.1371/journal.pcbi.1010881)
Supplement: Attachment — Submitted filename: Reviewer comments for PCOMPBIOL_bh_nt.docx [file pcbi.1010881.s016.docx]

We would like to thank the reviewers for their compliments and suggestions. We will be addressing them point-by-point below.

Reviewer #1: This paper demonstrated an interesting and useful approach for the SSN analysis, i.e., what is an appropriate e-value threshold to delineate different subfamilies (or subgroups). Indeed, there is no systematic method to solve this problem and researchers use arbitrary and intuitive cutoffs to generate own SSNs in general. The approach that the authors described in this paper offers a nice solution for this problem.

The paper flows well and well written in general. I believe that this paper has a great value to publish in PLoS Computational Biology while I have some suggestions to improve the manuscript.

1. While the authors presented the approach to measure several parameters for the SSNs, there is almost no description in the method. The general methods have been previously developed and the authors simply cite those papers in Methods. As this paper is really centered for the utility of the methodology, I request to describe step-by-step protocol that other researcher can replicate and adopt to other system. If they use own script, the authors must upload it to github to share it to the community.

This one is indeed a very valid criticism. We have uploaded on github (<https://github.com/bastian-wur/closeness_by_component>) an user-oriented script, which only requires a network file [. We also have added a section explaining the mathematics/principle of closeness centrality into the manuscript,](https://github.com/bastian-wur/closeness_by_component) lines 676-700.

It is always difficult/impossible to know if clustered sequences represent isofunctional subfamily or just clustered sequences (more subgourp) with potentially multiple sub-subfamilies. It is important to map and highlight sequences with experimentally characterized in the SSNs. I wonder if the authors can highlight those sequences for each family they have characterized. They can isolate those experimentally validated sequences from Uniprot and map onto their SSNs. Mapping those information gives much confidence for the utility of the approach.

This is a good point, but we found the highlighting of the experimentally validated enzymes in the networks a bit cluttering, especially on more complicated examples. We have instead uploaded the Cytoscape files to figshare under https://doi.org/10.6084/m9.figshare.21671717, including relevant metadata like the Uniprot identifiers of experimentally characterized enzymes, to allow the reader to visualize this data with an optimal tool. In addition, we have added more supplementary figures (now figure S2-4), showing the distribution of experimentally validated enzymes (EC numbers) in the larger subfamilies (GH45, GH55, GH68). In these new figures, we notably highlighted the representativeness of each enzymatic activity, with the number of characterized protein for each. Such information is indeed essential and will also appear on CAZy website for each family and subfamily in the close future.

Reviewer comments for PCOMPBIOL-D-22-00646

“An objective criterion to evaluate sequence-similarity networks helps in dividing the protein family sequence space”

Protein functional annotation is still dependent on expert curation for the most accurate delineation. The attached manuscript describes a novel use of closenesss centrality, a well-known measure in network analysis, to analyze sequence similarity networks (SSNs). The authors rather exhaustively test this criterion on artificial data, known protein families with existing network analyses, and novel families that have not yet been analyzed by SSN. Despite lacking the more rigorous evolutionary foundation of phylogenetic analysis, the authors make a strong case that SSNs are a more performant method for accounting for the deluge of sequencing data. This work is novel, has a strong premise, and is an innovation combination of well-established databases and graph network analyses. Nonetheless, there are a few points that should be addressed in order for this work to be published. I have organized them into major and minor points below. I recommend that this work be published after minor revision.

We would like to thank the reviewer for his kind words and appreciation of our work. It has been a nice experience to read and answer both reviews to improve our manuscript.

**Major Points**

1) A major strength of SSNs is their relatively perfomance with respect to phylogenetic analyses and other types of dimension reduction. Similarly, a stated value of the closeness centrality criterion is that it is faster than manually testing e-value cutoffs. I would like to see more explicit benchmarking data for the artificial data, known families, and novel families. If run times are neglible a statement in the methods to that effect would be appropriate. A table, either in the supplementary material or main text elaborating on this performance and how it varies with respect to family size would also work.

We indeed considered runtimes to be mostly negligible, but did a short benchmark on the smallest and the biggest of the CAZy networks we processed, and have added this in lines 715-630

2) The sizes and cutoffs of the various families being considered are difficult to follow in the text. It would provide clarity to make a summary table that elaborated on the families considered, their accessions in CAZY (where appropriate), the cutoffs determined by closeness centrality, and any cutoffs determined previousy in other works.

We agree that tracking all the results can be complicated, and have therefore added the requested table as supplementary table 3.

3) The current figures are not publication ready. Generally, even at full size, it is difficult to resolve edge nodes, axis labels are illegible. Insofar as a principal point of the manuscript is the relative ease of SSNs and their visualization, the complexity of these figures, both individually and collectively, works against this point. I would recommend a) making a schematic representation of the artificial data results and either making it comparative with the “real” data, then having a more legible version of the closeness vs. cutoff graph. I am also not certain the full set of these figures is necessary in the main text, some could be moved to supplementary.

We are sorry about these issues, we would guess that this might be an issue with the submission system, and not actually with our figures. To address these concerns (and also of reviewer 1), we have added the Cytoscape files and the full resolution figures to a figshare repository under <https://doi.org/10.6084/m9.figshare.21671717>, so that everyone can see everything as intended and can also investigate these networks.

4) I would like some more elaboration (perhaps in the form of a figure) of an example where the closeness criterion is separating into functional vs. structural groups

We included a sentence line 455-458 during the discussion about the multiple local optima. However, we did not elaborate deeply on this question which is complex. While this criterion allow to distinguish the major shifts in the sequence conservation, we have no definite idea if these separations are due to structural or to functional changes. While one could expect structural changes to be more “massive” and thus to arise early in family evolution (at high e-values thresholds), these changes could either induce changes in substrate preferences, as demonstrated by Murakami and coworkers on GH128 (PMID: **32451508)**, or not, as for example some extra-loops could increase enzymatic affinity/avidity without changing the target substrate (as we potentially expect for GH51). As stated in the GH19, “deeper subfamily splits result in a separation of structural features, and not of functions”. Similarly, functional differentiation could emerge early in a family evolution (after duplication and neofunctionalization) and will be capture by SSN thanks to numerous additional mutation along with each subfamily evolution, while only a few substitutions in the +1 or -1 catalytic sites might induce a functional shift, preventing SSN detection if not followed by additional changes, and leading to multifunctional subfamilies.

**Minor Points**

1) There are numerous grammatical errors. A close reading for line edits is necessary. I have provided some corrections below but the list is not exhaustive.

P2,L50:	Determining => Determine

P2,L52:	“Cazbohydrate”

P2,L54:	“And guide” => “And to guide”

P2,L99:	“build hypothesis” => “build hypotheses” or “build a hypothesis”

P6,L146:”consisted in network collections” => “consisted of network collections”

P6,L155: Not clear what i) ii) iii) iv) are indicating in this paragraph

P11,L334: “Such situation might” => “Such a situation might”

P12,L351: “group has not been seen” => “group had not been seen”

All these points have been fixed.
